# Supplementary material for: MetaQTL: a package of new computational methods for the meta-analysis of QTL mapping experiments
Source: BMC Bioinformatics. 2007 Feb 8;8:49. doi: 10.1186/1471-2105-8-49 (PMC1808479; doi:10.1186/1471-2105-8-49)
Supplement: Additional File 7 — MetaQTL Package : jar file and tutorial. This Zip archive contains both the MetaQTL JAR file and the files of the tutorial. [file 1471-2105-8-49-S7.zip › org.inra.metaqtl/doc/index-files/index-9.html]

I-Index


|  |  |  |
| --- | --- | --- |
| **Overview** \| Package \| Class \| Use \| **Tree** \| **Deprecated** \| **Index** \| **Help** | |  |
| **PREV LETTER**   **NEXT LETTER** | **FRAMES**    **NO FRAMES**     **All Classes** |  |


A B C D E F G H I K L M N O P Q R S T U V W X Y Z 

---


## **I**

**IBioAdaptable** - Interface in org.thalia.bio: **IBioAdapter** - Interface in org.thalia.bio: **IBioAllele** - Interface in org.thalia.bio: Class Description Here **IBioConstants** - Interface in org.thalia.bio.entity: Class Description Here **IBioCross** - Interface in org.thalia.bio: Class Description Here **IBioCrossTypes** - Interface in org.thalia.bio: Class Description Here **IBioEntity** - Interface in org.thalia.bio: Class Description Here **IBioEntityTypes** - Interface in org.thalia.bio: Class Description Here **IBioGenome** - Interface in org.thalia.bio: **IBioGenomeProperties** - Interface in org.metaqtl: This interface defines both the keys and the default values of properties which can be handled by a org.thalia.bio.entity.IBioGenome. **IBioIndividual** - Interface in org.thalia.bio: Class Description Here **IBioIndividualTree** - Interface in org.thalia.bio: Class Description Here **IBioLGroup** - Interface in org.thalia.bio: **IBioLGroupProperties** - Interface in org.metaqtl: This interface defines the key of properties which can be handled by a org.thalia.bio.entity.IBioLGroup. **IBioLocus** - Interface in org.thalia.bio: **IBioLocusProperties** - Interface in org.metaqtl: This interface defines the key of the properties which can be handled by a org.thalia.bio.entity.IBioLocus. **IBioOntology** - Interface in org.thalia.bio: This interface defines some rules to handle a ontology. **IBioOntologyTerm** - Interface in org.thalia.bio: This class defines a term of an ontology. **IBioPopulation** - Interface in org.thalia.bio: Class Description Here **ic1** - Variable in class org.metaqtl.Qtl.QTLSupport: The recombination rate between the QTL position and the left border of the IC. **ic2** - Variable in class org.metaqtl.Qtl.QTLSupport: The recombination rate between the QTL position and the right border of the IC. **ICOMP** - Static variable in class org.metaqtl.EMCriteria: **icomp** - Variable in class org.metaqtl.EMCriteria: **ICOMP\_NAME** - Static variable in class org.metaqtl.EMCriteria: **id** - Variable in class org.metaqtl.Qtl: The id of the Qtl **id** - Variable in class org.thalia.bio.entity.bean.OntologyTermBean: The id of the term. **idx** - Variable in class org.metaqtl.TreeNode: ? The identifiant of the cluster. **idx1** - Variable in class org.metaqtl.CMarkerSequence: The indices of the markers on the first chromosome for the common sequences. **idx2** - Variable in class org.metaqtl.CMarkerSequence: The indices of the marker on the second chromosome for the common sequences. **IGeneticMapProperties** - Interface in org.thalia.bio.entity: Class Description Here **ILGroupPosition** - Interface in org.thalia.bio.util: Class Description Here **IMetaAlgorithm** - Interface in org.metaqtl: This interface defines some methods which are common to data analysis algorithms. **IMetaQtlConstants** - Interface in org.metaqtl: This interface defines some global constants which are used by several classes of the package. **IMIN(int, int)** - Static method in class org.metaqtl.numrec.NumericalUtilities: Returns the min between `a` and `b` **incms** - Variable in class org.metaqtl.CMarkerSequence: **indexx(int, double[], int[])** - Static method in class org.metaqtl.numrec.NumericalUtilities: Sort the given array of double arrin[1..n] without destroying it and returns the sorted indices of the array elements into indx[1..n]. **Individual** - Class in org.thalia.bio.entity: Class Description Here **Individual()** - Constructor for class org.thalia.bio.entity.Individual: **Individual(String, IBioEntity)** - Constructor for class org.thalia.bio.entity.Individual: **INDIVIDUAL** - Static variable in interface org.thalia.bio.IBioEntityTypes: The integer code for an individual entity **IndividualContainer** - Class in org.thalia.bio.entity: Class Description Here **IndividualContainer()** - Constructor for class org.thalia.bio.entity.IndividualContainer: **IndividualContainer(String, IBioEntity)** - Constructor for class org.thalia.bio.entity.IndividualContainer: **individuals()** - Method in class org.thalia.bio.entity.IndividualContainer: **individuals()** - Method in interface org.thalia.bio.IBioPopulation: **InfoMap** - Class in org.metaqtl.main: **InfoMap()** - Constructor for class org.metaqtl.main.InfoMap: **init(Graphics2D, ChromGraph[])** - Method in class org.metaqtl.graph.MetaGraph: **initCmdLineParser()** - Method in class org.metaqtl.main.MetaMain: **initEM(double[], double[], EMResult, Random)** - Static method in class org.metaqtl.algo.EMAlgorithm: Initializes the EM algorithm by randomly assigning observations to the clusters and do one M-Step to compute the first values of the parameters. **initNodeIdx()** - Method in class org.metaqtl.Tree: **initPalettes()** - Static method in class org.metaqtl.graph.MetaGraphPar: **initProbaPalette()** - Static method in class org.metaqtl.graph.MetaGraphPar: **initQTLPalette()** - Static method in class org.metaqtl.graph.MetaGraphPar: **initQTLPalette(int)** - Static method in class org.metaqtl.graph.MetaGraphPar: **initReverse()** - Method in class org.metaqtl.Tree: **intScale** - Variable in class org.metaqtl.Qtl.QTLProj: The scale ratio between the original flanking marker interval and the one on the map used for projection. **IOntologyTermProperties** - Interface in org.thalia.bio: **IRISe** - Static variable in interface org.thalia.bio.IBioCrossTypes: **IRISi** - Static variable in interface org.thalia.bio.IBioCrossTypes: **isAssignableEntities(IResource, IBioEntity[])** - Static method in class org.thalia.bio.util.ResourceBioEntityQuery: Returns true if all elements of entities are included in the resource. **isAssignableEntity(IResource, IBioEntity)** - Static method in class org.thalia.bio.util.ResourceBioEntityQuery: Returns true if the resource contains the given entity. **isConnected(int)** - Method in class org.metaqtl.ChromCluster.ClusterGraph: Test if the graph is connected **isConnected(int)** - Method in class org.metaqtl.ChromCluster: Tests if the cluster of chromosome is connected using a graph approach. **isDubiousLocus(IBioLocus)** - Method in class org.metaqtl.ChromCluster: If a list of dubious markers has been set before then this routine checks if the given locus is not included into this list. **isDubiousOrderLocus(IBioLocus)** - Static method in class org.metaqtl.util.LocusProperties: Returns true if the given locus has a property order.dubious set to one, false otherwise **isIgnore()** - Method in class org.metaqtl.Qtl: This methods returns true if the QTL must be ignored. **isLoggerEnable()** - Method in class org.metaqtl.algo.MetaAlgorithm: **ISWAP(int[], int, int)** - Static method in class org.metaqtl.numrec.NumericalUtilities: Swaps elements i and j in a. **iterate(double[], double[], EMResult)** - Static method in class org.metaqtl.algo.EMAlgorithm: This method performs one iteration of the EM-algorithm.

---


|  |  |  |
| --- | --- | --- |
| **Overview** \| Package \| Class \| Use \| **Tree** \| **Deprecated** \| **Index** \| **Help** | |  |
| **PREV LETTER**   **NEXT LETTER** | **FRAMES**    **NO FRAMES**     **All Classes** |  |


A B C D E F G H I K L M N O P Q R S T U V W X Y Z 

---
